# *SMALL GRAIN 11* Controls Grain Size, Grain Number and Grain Yield in Rice

**DOI:** 10.1186/s12284-016-0136-z

**Published:** 2016-11-29

**Authors:** Na Fang, Ran Xu, Luojiang Huang, Baolan Zhang, Penggen Duan, Na Li, Yuehua Luo, Yunhai Li

**Affiliations:** 1 Hainan Key Laboratory for Sustainable Utilization of Tropical Bioresources/Agricultural College, Hainan University, Haikou, 570228 People’s Republic of China; 2 State Key Laboratory of Plant Cell and Chromosome Engineering, Institute of Genetics and Developmental Biology, Chinese Academy of Sciences, Beijing, 100101 People’s Republic of China

**Keywords:** Rice, *SMG11*/*D2*, Grain size, Cell expansion

## Abstract

**Background:**

Grain size is one of key agronomic traits that determine grain yield in rice. Several regulators of grain size have been identified in rice, but the mechanisms that determine grain size and yield remain largely unknown.

**Results:**

Here we characterize a *small grain* (*smg11*) mutant in rice, which exhibits small grains, dense panicles and the increased number of grains per panicle. Cloning and sequence analyses of the *SMG11* gene reveal that *smg11* is a new allele of *DWARF2* (*D2*), which encodes a cytochrome P450 (CYP90D2) involved in brassinosteroid biosynthetic pathway. Overexpression of *D2/SMG11* increases grain size and grain weight of wild-type plants. Overexpression of *D2/SMG11* at a suitable level also significantly increases grain yield in rice. Cellular analyses indicate that *D2/SMG11* controls grain size by promoting cell expansion. Further results reveal that *D2/SMG11* influences expression of several known grain size genes involved in the regulation of cell expansion, revealing a novel link between *D2/SMG11* and known grain size genes.

**Conclusions:**

*SMG11* controls grain size by promoting cell expansion in grain hulls. *SMG11* regulates cell expansion, at least in part, by influencing expression of several grain size genes involved in the regulation of cell expansion. The *smg11* is a new allele of *DWARF2/D2.* The suitable expression of *SMG11* increases grain size, grain weight and grain yield. Our findings reveal the functions of *D2/SMG11* in grain size and grain yield, suggesting that the suitable expression of *D2/SMG11* is a promising approach to improve grain yield in rice.

**Electronic supplementary material:**

The online version of this article (doi:10.1186/s12284-016-0136-z) contains supplementary material, which is available to authorized users.

## Background

Rice is one of the most important cereal crops and the main food source of the global population. Grain yield is coordinately controlled by grain weight, grain number per panicle, panicle number per plant, and grain filling ratio. Grain weight is positively associated with grain size, which is determined by grain length, grain width, and grain thickness. Several factors that regulate grain size have been reported in rice (Zuo and Li, 2014; Li and Li, 2016), but the genetic and molecular mechanisms of grain size control remain largely unknown.

Several factors that regulate grain size by influencing cell proliferation have been described in rice. The major QTL for both grain length and grain weight (*GS3*) encodes a putative G protein γ subunit and negatively regulates grain size (Fan et al., 2006; Mao et al., 2010). However, its Arabidopsis homolog (*AGG3*) promotes seed and organ growth by increasing cell proliferation (Li et al., 2012). The major QTL for grain length (*qGL3/GL3.1*), which encodes a putative protein phosphatase with Kelch-like repeat domain (OsPPKL1), influences grain length by limiting cell proliferation (Hu et al., 2012; Qi et al., 2012; Zhang et al., 2012). The major QTL for grain width and weight (*GW2*) encodes a RING-type E3 ubiquitin ligase, which restricts cell proliferation in spikelet hulls (Song et al., 2007). Arabidopsis DA2 shares significant similarity with rice GW2 and functions as a negative regulator of seed and organ size in Arabidopsis (Xia et al., 2013). A ubiquitin-related protein encoded by rice *SEED WIDTH ON CHROMOSOME 5* (*qSW5/GW5*) influences grain width by limiting cell proliferation (Shomura et al., 2008; Weng et al., 2008). Higher expression of *GW8*/*OsSPL16* promotes cell division and grain filling, resulting in wide and heavy grains in rice (Wang et al., 2012). *GS5*, which encodes a putative serine carboxypeptidase, promotes cell proliferation in spikelet hulls (Li et al., 2011). The MAPK pathway has recently reported to control grain size by increasing cell proliferation in spikelet hulls (Duan et al., 2014; Liu et al., 2015). In addition, several genes that regulate grain size by influencing cell expansion have been reported in rice. The major QTL for grain size (*GS2*) encodes Growth-Regulating Factor 4 (OsGRF4) (Che et al., 2015; Duan et al., 2015; Hu et al., 2015). OsGRF4/GS2 physically interacts with transcriptional coactivators GRF-interacting Factors (GIFs) to regulate grain size by increasing both cell expansion and cell proliferation in spikelet hulls (Duan et al., 2015). The major QTL for grain length and width (*GLW7*) encodes the transcription factor OsSPL13, which promotes grain growth by increasing cell expansion in spikelet hulls (Si et al., 2016). OsSPL13 associates the promoter of *SMALL AND ROUND SEEDS* (*SRS5*) and regulates its expression (Si et al., 2016). The bHLH transcription factors PGL1 and PGL2 promote grain growth by increasing cell expansion in spikelet hulls, while the bHLH transcription factor APG functions antagonistically with PGL1 and PGL2 to influence grain length (Heang and Sassa, 2012a, b). Thus, the transcriptional regulatory factors play important roles in rice grain size control. Several recent studies show that elevated expression of *GL7/GW7/SLG7*, which encodes a protein homologous to *Arabidopsis thaliana* LONGIFOLIA1/2, produces slender and long grains (Wang et al., 2015a; Wang et al., 2015b; Zhou et al., 2015). GL7/GW7/SLG7 has been reported to affect grain length and shape by increasing cell elongation in spikelet hulls (Wang et al., 2015b; Zhou et al., 2015), while another study show that GL7/GW7/SLG7 controls grain size by influencing cell proliferation in spikelet hulls (Wang et al., 2015a). Thus, grain size is coordinately determined by cell proliferation and cell expansion in rice.

To further understand the molecular mechanisms that determine grain size, we have previously isolated *small grain mutants* (*smg*) in rice (Duan et al., 2014). Here we report that *smg11* is a new allele of *DWARF2* (*D2*), which encodes a cytochrome P450 (CYP90D2) involved in brassinosteroid (BR) biosynthetic pathway (Hong et al., 2005). Brassinosteroids play important roles in plant growth and development. Several studies show that genes involved in BR signaling and BR biosynthetic pathways influence seed size in Arabidopsis and rice (Zuo and Li, 2014; Li and Li, 2015, 2016). In this study, our results reveal that SMG11/DWARF2 (D2) positively regulates grain size by promoting cell expansion in spikelet hulls. *smg11* mutant produces small grains due to decreased cell expansion, while overexpression of *SMG11* causes large grains as a result of increased cell expansion. Further results show that *SMG11* affects expression of several known grain size genes involved in the regulation of cell expansion, revealing a novel link between D2/SMG11 and known grain size regulators. A suitable expression of *SMG11* also significantly enhances grain yield in rice. Thus, our findings identify the functions of *SMG11* in grain size and yield control and give insight into how grain size is determined in rice.

## Results

### *smg11* Produces Small Grains

**Fig. 1:**
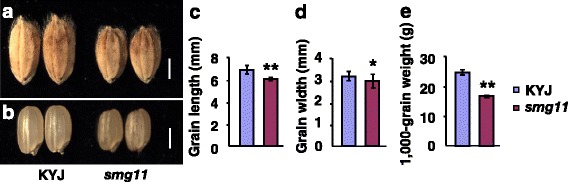
The *smg11* mutant produces small grains. **a** Mature paddy rice grains of KYJ and *smg11*. **b** Brown rice grains of KYJ and *smg11*. **c** The average length of KYJ and *smg11* grains. **d** The average width of KYJ and *smg11* grains. **e** The 1000-grain weight of KYJ and *smg11*. Values **c**-**e** are given as mean ± SD. *P < 0.05; **P < 0.01 compared with the wild type by Student’s *t*-test. Bars: 2 mm in **a** and **b**

To understand genetic and molecular mechanisms that set the final size of grains, we have previously identified mutants with altered grain size in rice (Duan et al., 2014). The *small grain 11* (*smg11*) mutant was isolated from the ethyl methanesulfonate (EMS)-treated M_2_ populations of the *japonica* variety Kuanyejing (KYJ). The *smg11* mutant showed obviously smaller grains than KYJ (Fig. 1a, b). The length and width of *smg11* grains was significantly decreased compared with that of KYJ grains (Fig. 1c, d). The average length of KYJ and *smg11* grains was 6.93 mm and 6.03 mm, respectively. By contrast, the average width of KYJ and *smg11* grains was 3.16 mm and 2.95 mm, respectively. In addition, the 1000-grain weight of *smg11* was dramatically reduced compared with that of the wild type (Fig. 1e). The 1, 000-grain weight of KYJ was 24.93 g, while the 1, 000-grain weight of *smg11* was only 16.86 g. Therefore, these results indicate that the *smg11* mutation influences grain size and weight in rice.

### *smg11* Forms Dense and Erect Panicles With Increased Grain Number

**Fig. 2:**
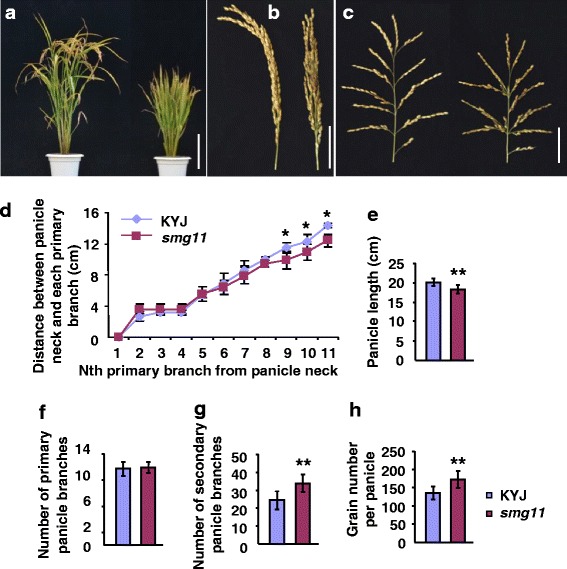
*smg11* produces dense and erect panicles. **a** KYJ (*left*) and *smg11* (*right*) plants at the mature stage. **b c** Panicles of KYJ (*left*) and *smg11* (*right*) at the mature stage. **d** Distance between panicle neck and each primary branch of KYJ and *smg11* panicles. **e** The average length of KYJ and *smg11* panicles. **f** Total number of KYJ and *smg11* primary branches. **g** Total number of KYJ and *smg11* secondary branches. **h** Grain number per panicle in KYJ and *smg11*. Values **d**-**h** are given as the mean ± SD. *P < 0.05; **P < 0.01 compared with the wild type by Student’s *t*-test. Bars: 20 cm in **a**; 5 cm in **b**-**c**

The *smg11* plants were obviously shorter than wild-type plants (Fig. 2a, e). The *smg11* leaves were more erect than wild-type leaves (Fig. 2a). At the mature stage, the panicles of *smg11* exhibited the erect phenotype compared with KYJ panicles (Fig. 2b). The panicles of *smg11* were also shorter and denser than those of the wild type (Fig. 2b–c). The panicle axis of *smg11* was slightly shorter than that of the wild type (Fig. 2d). These results indicate that the *smg11* mutation affects panicle size and shape. We then counted the number of primary and secondary panicle branches. As shown in Fig. 2f, g, the number of primary panicle branches in *smg11* was similar to that in KYJ, while the number of secondary panicle branches in *smg11* was significantly increased in comparison to that in KYJ. We also observed that the grain number per panicle in *smg11* was higher than that in KYJ (Fig. 2c, h). Thus, these results show that the dense panicle phenotype of *smg11* was due to a decrease in the length of panicle axis and increases in the secondary panicle branch number and grain number per panicle.

### *smg11* Decreases Cell Expansion in Spikelet Hulls and Influences Expression of Several Known Grain Size Genes

**Fig. 3:**
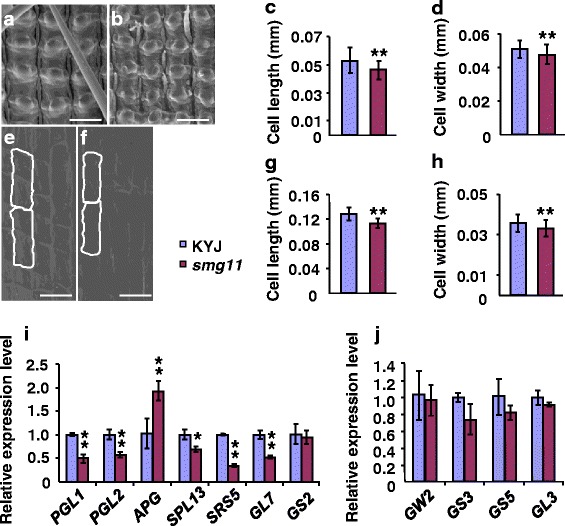
*smg11* influences cell expansion. **a b** SEM analysis of the outer surface of KYJ **a** and *smg11* **b** lemmas. **c** The average length of outer epidermal cells in KYJ and *smg11* lemmas. **d** The average width of outer epidermal cells in KYJ and *smg11* lemmas. **e f** SEM analysis of the inner surface of KYJ **e** and *smg11* **f** lemmas. **g** The average length of inner epidermal cells in KYJ and *smg11* lemmas. **h** The average width of inner epidermal cells in KYJ and *smg11* lemmas. **i** Expression levels of the indicated genes in KYJ and *smg11* panicles. **j** Expression levels of the indicated genes in KYJ and *smg11* panicles. Values **c**-**d**; **g**–**j** are means ± SD. *P < 0.05; **P < 0.01 compared with the wild type by Student’s *t*-test. Bars: 100 μm in **a**, **b**, **e**, **f**

The size of a grain has been known to be restricted by its spikelet hull, which may set an upper limit to final grain size (Li and Li, 2016). The growth of spikelet hulls is coordinately determined by cell proliferation and cell expansion. We therefore examined cells in KYJ and *smg11* spikelet hulls. As shown in Fig. 3a–d, outer epidermal cells in *smg11* spikelet hulls were significantly shorter and narrower than those in KYJ. Similarly, inner epidermal cells in *smg11* spikelet hulls were shorter and narrower than those in KYJ spikelet hulls (Fig. 3e–h). By contrast, the number of epidermal cells in the grain-length direction in *smg11* spikelet hulls was similar to that in KYJ spikelet hulls (Additional file 1: Figure S4). These results indicate that the small grain phenotype of *smg11* mainly results from the reduced cell expansion in spikelet hulls.

To understand how *D2/SMG11* regulates cell expansion in spikelet hulls, we investigated expression of several known grain size genes involved in the regulation of cell expansion, including *GS2*, *GL7*, *GLW7/SPL13, PGL1, PGL2, APG* and *SRS5*. The transcription factor SPL13/GWL7 has been recently reported to promote expression of *SRS5* that encodes alpha-tubulin protein (Si et al., 2016). Overexpression of *SPL13* increased grain length by promoting cell elongation in spikelet hulls (Si et al., 2016). Expression levels of both *SPL13/GWL7* and *SRS5* in *smg11* panicles were lower than those in KYJ panicles (Fig. 3i). The bHLH transcription factors PGL1 and PGL2 positively regulate grain length by increasing cell expansion in spikelet hulls (Heang and Sassa, 2012a, b). In contrast, the bHLH transcription factor APG functions antagonistically with PGL1 and PGL2 to influence grain length by influencing cell expansion in spikelet hulls (Heang and Sassa, 2012a, b). As shown in Fig. 3i, expression levels of *PGL1* and *PGL2* in *smg11* were lower than those in KYJ, while expression level of *APG* in *smg11* was higher than that in KYJ. Two studies showed that *GL7/GW7/SLG7* regulates grain size by influencing cell expansion in spikelet hulls (Wang et al., 2015b; Zhou et al., 2015), although another study reported that *GL7/GW7/SLG7* controls grain size by regulating cell proliferation (Wang et al., 2015a). We observed that expression of *GL7/GW7/SLG7* was significantly reduced in s*mg11* in comparison to that in KYJ (Fig. 3i). The transcription factor GS2 and a putative serine carboxypeptidase GS5 regulate grain size by promoting both cell proliferation and cell expansion (Li et al., 2011; Che et al., 2015; Duan et al., 2015; Hu et al., 2015). Expression levels of *GS2* and *GS5* in *smg11* were similar to those in KYJ (Fig. 3i, j). Furthermore, we detected expression of several grain size genes involved in the regulation of cell proliferation in spikelet hulls, including *GW2*, *GS3* and *GL3*. As shown in Fig. 3j, expression levels of *GW2*, *GS3* and *GL3* in *smg11* were not strongly altered in comparison to those in KYJ, further suggesting the role of *SMG11* in cell expansion. Taken together, these results suggest that *SMG11* regulates grain size, at least in part, by influencing expression of these grain size genes involved in the regulation of cell expansion.

### *smg11* is a Novel Allele of the *DWARF 2* (*D2*) Gene

The *smg11* mutation was identified using the MutMap approach (Abe et al., 2012), which is based on whole-genome resequencing of bulked DNA of F2 segregants. An F2 population of a cross between *smg11* and the parental line KYJ were generated. The segregation of F2 progenies showed that the phenotypes of *smg11* are determined by a single recessive gene. DNA from 50 F2 individuals that showed the small-grain phenotype was pooled in an equal ratio and subjected to whole-genome resequencing, and DNA from KYJ was resequenced as a control. We obtained a total of 5.6 Gbp of short reads for KYJ and 8.7 Gbp for the bulked F2 plants. 2928 SNPs and 423 INDELs that represent the polymorphisms between the bulked F2 and KYJ were detected. For these SNPs and INDELs, the SNP/INDEL-index (the ratio between the number of reads of a mutant SNP/INDEL and the total number of reads corresponding to the SNP/INDEL) were calculated. The causative SNP/INDEL should be shared by all the mutant F2 plants and therefore has a SNP/INDEL-index = 1. In all, 47 SNPs and 34 INDELs have a SNP/INDEL-index = 1, while only 2 SNPs were identified in exons (Additional file 1: Figure S1). The SNP1 represents a missense mutation of a proline (CCC) codon to a leucine (CTC) in *LOC_Os01g10040*, while the SNP2 is corresponded to a synonymous mutation. Thus, these results suggest that *LOC_Os01g10040* is a good candidate gene for *SMG11*.

**Fig. 4:**
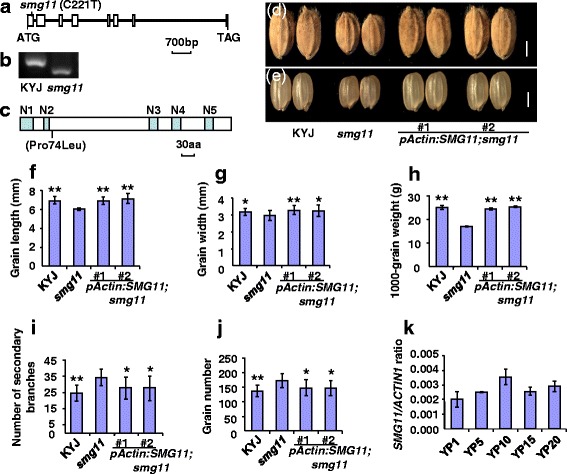
Identification of the *D2/SMG11* gene. **a** The *D2/SMG11* gene structure. The white boxes indicate the exons, and black lines indicate the introns. The start codon (ATG) and the stop codon (TAG) are indicated. The *smg11* mutation in the *SMG11/D2* gene is shown. **b** The dCAPS marker is developed according to the *smg11* mutation. **c** The D2/SMG11 protein contains a membrane anchor domain (N1), a proline-rich domain (N2), a dioxygen binding domain (N3), a steroid binding domain (N4) and a heme binding domain (N5). **d e** Grains of KYJ, *smg11*, *pActin*: *SMG11*;*smg11#1* and *pActin*: *SMG11*;*smg11#2. pActin*: *SMG11*;*smg11* is *smg11* transformed with *pActin*: *SMG11*. **f g h** Grain length **f** grain width **g** and 1000-grain weight **h** of KYJ, *smg11*, *pActin*: *SMG11*;*smg11#1* and *pActin*: *SMG11*;*smg11#2*. **i** The number of KYJ, *smg11*, *pActin*: *SMG11*;*smg11#1* and *pActin*: *SMG11*;*smg11#2* secondary panicle branches. **j** Grain number per panicle of KYJ, *smg11*, *pActin*: *SMG11*;*smg11#1* and *pActin*: *SMG11*;*smg11#2*. **k** Quantitative real-time RT-PCR analysis of *SMG11/D2* gene expression in developing panicles of 1 cm (YP1) to 20 cm (YP20). Values **f**–**k** are given as mean ± SD. *P < 0.05; **P < 0.01 compared with *smg11* by Student’s *t*-test. Bars: 2 mm in **d**-**e**

The identity of the *SMG11* gene was further confirmed by genetic complementation analysis. A plasmid that contained wild-type gene of *LOC_Os01g10040* driven by a *Actin* promoter (*pActin: SMG11*) was introduced into the *smg11* mutant. We generated 58 transgenic plants and found that transgenic plants showed wild-type phenotypes (Fig. 4d–j). For example, grain length, grain width and 1000-grain weight of *pActin: SMG11;smg11* were similar to those of KYJ. Similarly, the secondary branch number and grain number per panicle of *pActin: SMG11;smg11* were comparable with those of KYJ. In addition, *pActin: SMG11;smg11* transgenic lines exhibited similar plant height and panicle length to KYJ plants (Additional file 1: Figure S2). Therefore, these results indicate that *LOC_Os01g10040* is the *SMG11* gene.

The gene *LOC_Os01g10040* encodes a cytochrome P450 protein CYP90D2/DWARF2 (D2), which is involved in the BR biosynthesis pathway (Hong et al., 2005). Expression of *SMG11/D2* was detected in developing panicles (Fig. 4k), consistent with the roles of *D2/SMG11* in grain size and panicle size control. The *smg11* has a C to T transition in codon 74 (CCC/CTC) of *LOC_Os01g10040* (Fig. 4a, c), resulting in a Pro/Leu amino acid substitution (Fig. 4c). Sequence alignment of several rice and Arabiodpsis SMG11/D2 homologs showed that Pro in the position 74 is a conserved amino acid (Additional file 1: Figure S3). These results show that *smg11* is a novel allele of *CYP90D2/D2*.

### *smg11* Affects Expression of Brassinosteroid Biosynthetic and Signaling Genes

**Fig. 5:**
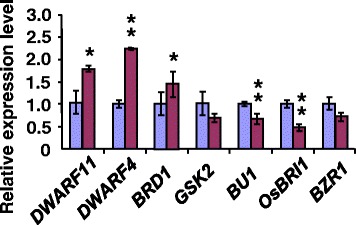
Expression levels of BR-related genes in KYJ and *smg11* panicles. Quantitative real-time RT-PCR analysis of BR-related gene expression in young panicles. Values are means ± SD. *P < 0.05 **P < 0.01 compared with the wild type by Student’s *t*-test

It is known that BR signaling mutants or BR-synthetic mutants influence expression levels of BR-synthetic genes as a feedback mechanism (Tong et al., 2009; Duan et al., 2014). We therefore examined expression levels of several BR-synthetic genes in KYJ and *smg11* panicles, including *DWARF4*, *DWARF11* and *BRD1* (Hong et al., 2002; Mori et al., 2002; Tanabe et al., 2005; Sakamoto et al., 2006)*.* As shown in Fig. 5, expression levels of *DWARF4*, *DWARF11* and *BRD1* in *smg11* panicles were higher than those in KYJ panicles. It is possible that the mutation in *SMG11/D2* resulted in the feedback up-regulation of BR biosynthetic genes in rice.

We then asked whether *SMG11* influences expression of BR-signaling genes involved in the regulation grain size, such as *OsBRI1*, *GSK2*, *OsBZR1*, and *BU1* (Yamamuro et al., 2000; Tanaka et al., 2009; Tong et al., 2012; Zhu et al., 2015). We performed quantitative real-time RT-PCR analysis to investigate their expression levels in KYJ and *smg11* panicles. As shown in Fig. 5, expression levels of *BRI1* and *BU1* in *smg11* panicles were lower than those in KYJ panicles, while expression levels of *GSK2* and *OsBZR1* in *smg11* panicles were not significantly different from those in KYJ panicles. It is plausible that the mutation in *SMG11/D2* might decrease BR signaling or responses by repressing expression of BR signaling genes (e.g. *OsBRI1* and *BU1*), resulting in small grains.

### Overexpression of *SMG11* Increases Grain Size due to Large Cells in Spikelet Hulls

**Fig. 6:**
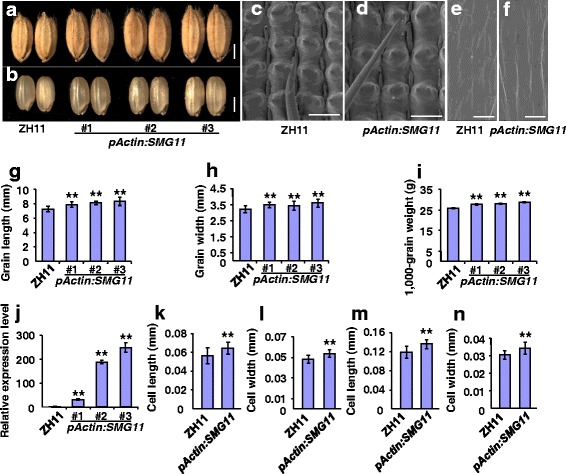
Overexpression of *D2/SMG11* increases grain size due to large cells in spikelet hulls. **a b** Grains of ZH11, *pActin*: *SMG11#1*, *pActin*: *SMG11#2* and *pActin*: *SMG11#3. pActin*: *SMG11* is ZH11 transformed with *pActin*: *SMG11*. **c d** SEM analysis of the outer surface of ZH11 **c** and *pActin*: *SMG11#2* **d** lemmas. **e**, **f** SEM analysis of the inner surface of ZH11 **e** and *pActin*: *SMG11#2* **f** lemmas. **g h i** Grain length **g** grain width **h** and 1000-grain weight **i** of ZH11, *pActin*:*SMG11#1*, *pActin*:*SMG11#2* and *pActin*:*SMG11#3*. **j** Expression levels of *SMG11/D2* in ZH11, *pActin*:*SMG11#1*, *pActin*:*SMG11#2* and *pActin*:*SMG11#3* panicles. **k** The average length of outer epidermal cells in ZH11 and *pActin*:*SMG11#2* lemmas. **l** The average width of outer epidermal cells in ZH11 and *pActin*:*SMG11#2* lemmas. **m** The average length of inner epidermal cells in ZH11 and *pActin*:*SMG11#2* lemmas. **n** The average width of inner epidermal cells in ZH11 and *pActin*:*SMG11#2* lemmas. Values **g**–**n** are given as mean ± SD. *P < 0.05 **P < 0.01 compared with ZH11 by Student’s *t*-test. Bars: 2 mm in **a**-**b** 100 μm in **c**–**f**

To further understand roles of *SMG11* in grain size control and its potential application in rice yield improvement, we expressed the *SMG11* gene driven by a *Actin* promoter (*pActin: SMG11*) in a *japonica* variety Zhonghua 11 (ZH11) and generated 29 transgenic plants. Transgenic plants produced longer and wider grains than ZH11 (Fig. 6a, b, g, h). The 1000-grain weight in *pActin: SMG11* transgenic plants was also significantly increased compared with that in ZH11 (Fig. 6i). We further examined expression levels of *SMG11/D2* in these transgenic plants (Fig. 6j). Expression levels of *SMG11/D2* were positively correlated with the grain size and weight phenotypes of transgenic plants.

To investigate cellular basis for the large grain phenotype of *pActin: SMG11* transgenic plants, we examined cell size of ZH11 and *pActin: SMG11* spikelet hulls. As shown in Fig. 6c, d, k, l, outer epidermal cells in *pActin: SMG11* spikelet hulls were significantly longer and wider than those in ZH11. Similarly, inner epidermal cells in *pActin: SMG11* spikelet hulls were longer and wider than those in ZH11 spikelet hulls (Fig. 6e, f, m, n). These results indicate that *SMG11* promotes grain growth by increasing cell expansion in spikelet hulls.

### A Suitable Expression of *SMG11* Increases Grain Yield in Rice

**Fig. 7:**
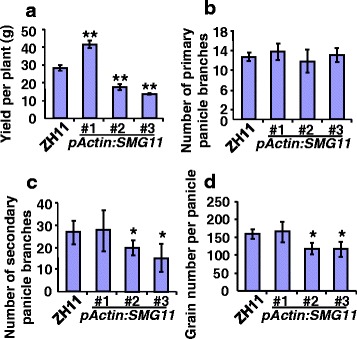
The suitable expression of *SMG11* increases grain yield. **a** Grain yield per plant of ZH11, *pActin*:*SMG11#1*, *pActin*:*SMG11#2* and *pActin*:*SMG11#3*. **b**–**d** The primary panicle branch number **b** the secondary panicle branch number **c** and grain number per panicle **d** of ZH11, *pActin*:*SMG11#1*, *pActin*:*SMG11#2* and *pActin*:*SMG11#3*. Values **a**–**d** are given as mean ± SD. *P < 0.05 **P < 0.01 compared with ZH11 by Student’s *t* test

As *pActin: SMG11* transgenic plants produced large and heavy grains, we asked whether overexpression of *SMG11* could increase grain yield in rice. We therefore investigated grain yield per plant of *pActin: SMG11* transgenic plants with different expression levels of *SMG11*. As shown in Fig. 6j, relative expression levels of *SMG11* in *pActin: SMG11#1, pActin: SMG11#2* and *pActin: SMG11#3* transgenic plants were 31.25, 177.42 and 246.66 folds higher than those in ZH11, respectively. *pActin: SMG11#1* transgenic plants exhibited higher yield per plant than ZH11 plants (Fig. 7a). In contrast, *pActin: SMG11#2* and *#3* transgenic plants decreased grain yield per plant (Fig. 7a), although they produced large and heavy grains (Fig. 6g–i). These results suggest that the effect of *D2/SMG11* on grain yield depends on its expression levels. To address why different *pActin: SMG11* lines showed opposite effects on grain yield, we investigated panicle branch number and grain number per panicle of *pActin: SMG11* transgenic lines. As shown in Fig. 7b–d, the primary panicle branch number, the secondary panicle branch number and grain number per panicle in *pActin: SMG11#1* were similar to those in ZH11, while the secondary panicle branch number and grain number per panicle in *pActin: SMG11#2* and *pActin: SMG11#3* were significantly reduced in comparison to those in ZH11. Thus, *pActin: SMG11#1* plants had normal panicle branch number as well as large and heavy grains, resulting in high grain yield. These results suggest that optimizing *SMG11* expression could be utilized to increase grain yield in rice.

## Discussion

Grain size is one of important agronomic traits in crops. Grain size is determined by grain length, grain width and grain thickness. Several factors that control grain size have been identified in rice, but the mechanisms that control grain size remain largely unknown. It is also a great challenge to improve rice yield using these grain size regulators. In this study, we report that *smg11* is a novel allele of *D2* (Hong et al., 2003). Our results show that *SMG11* promotes grain growth by increasing cell expansion in spikelet hulls. *SMG11* controls grain size, at least in part, by influencing expression of several known grain size genes involved in the regulation of cell expansion. Further results reveal that a suitable expression of *SMG11* increases grain size, grain weight and grain yield, suggesting that it is a promising target for rice yield improvement.

The *smg11* mutant produced small grains and dense, erect and short panicles, indicating that *SMG11* influences grain and panicle size. The secondary panicle branches and grain number per panicle in *smg11* were increased compared with KYJ, suggesting a possible balance mechanism between grain size and grain number. The *smg11* mutation occurred in the cytochrome P450 *CYP90D2/D2*, which is involved in BR biosynthesis (Hong et al., 2005). *smg11* is a novel allele of *D2/CYP90D2.* The *smg11* also showed similar phenotypes to BR defective mutants, such as reduced leaf angle and small grains (Additional file 1: Figures S5 and S6a). Exogenous application of BL rescued the leaf angle phenotype of *smg11* (Additional file 1: Fig. S6), further suggesting that the *smg11* mutation influences BR biosynthesis. Although several rice *d2* alleles have been previously reported (Hong et al., 2003; Hong et al., 2005; Li et al., 2013), none of these alleles was reported to increase the panicle branches and grain number per panicle. Consistent with the role of *SMG11* in panicle branch and grain number control, expression of *SMG11* complemented the panicle branch and grain number phenotypes of *smg11,* and strong expression of *SMG11* decreased panicle branches and grain number per panicle.

The *d2* alleles have been shown to produce small grains (Hong et al., 2003; Hong et al., 2005; Li et al., 2013), but how *D2* influences grain size is almost unknown. Our results showed that *smg11* had small grains due to short and narrow cells in spikelet hulls, indicating that *SMG11* regulates grain size by promoting cell expansion in both grain-length and grain-width directions. Several factors that control grain size by regulating cell expansion have been reported in rice. For example, the transcription factor SPL13/GWL7 binds to the promoter regions of *SRS5* and promotes its expression (Si et al., 2016). Overexpression of *SPL13* increased grain length by promoting cell elongation in spikelet hulls. Interestingly, we found that expression levels of both *SPL13/GWL7* and *SRS5* in *smg11* panicles were decreased compared with those in KYJ panicles. Elevated expression of *GL7*, which might be caused by increased copy number or mutations in the promoter, caused long grains due to increased cell elongation in spikelet hulls (Wang et al., 2015b; Zhou et al., 2015), although another study showed that *GL7* promotes cell proliferation in the grain-length direction (Wang et al., 2015a). Interestingly, the *smg11* mutation also reduced the expression of *GL7*. The bHLH transcription factors PGL1 and PGL2 have been known to positively regulate grain length by increasing cell expansion in spikelet hulls (Heang and Sassa, 2012a, b). In contrast, the bHLH transcription factor APG functions antagonistically with PGL1 to influence grain length (Heang and Sassa, 2012a, b). Consistent with the roles of PGL1, PGL2 and APG, the *smg11* mutation decreased expression of *PGL1* and *PGL2* and increased expression of *APG. D2/SMG11* has been known to be involved in BR biosynthesis (Hong et al., 2005). Several BR mutants have been reported to form small grains in rice, suggesting that BRs play key roles in grain size control. It is possible that BRs might regulate grain size by influencing expression of these grain size genes involved in the regulation of cell expansion, such as *SPL13, GL7, PGL1, PGL2* and *APG*. Consistent with this notion, PGL1 and APG have been proposed to influence BR signaling pathway (Heang and Sassa, 2012a, b). It will be interesting to test whether other BR-deficient mutants might affect expression of these grain size genes in the future.

BRs have been proposed to have potential applications in improving crop yields. In rice, a brassinosteroid-deficient mutant *osdwarf4-1* was reported to be associated with the increased grain yield under conditions of dense planting, even without extra fertilizer (Sakamoto et al., 2006). Expression of *OsDWARF4* driven by a specific promoter also increased grain yield in rice (Wu et al., 2008). In contrast, overexpression of *OsDWARF4* driven by a constitutive promoter caused defects in plant growth, resulting in a decrease in grain production. Therefore, it is possible that an optimized expression of BR-related genes could increase grain yield in rice. In this study, we found that overexpression of *SMG11* increased grain size and weight of wild-type plants, supporting that *SMG11* is a positive factor of gain size and weight and also suggesting that *SMG11* might be a good target for rice yield improvement. As we expected, *pActin: SMG11#1* transgenic plants with a suitable level of *SMG11* expression increased grain yield per plant. However, *pActin: SMG11#2* and *pActin: SMG11#3* transgenic plants with much strong expression of *SMG11* reduced grain yield due to decreases in panicle branches and grain number per panicle. Therefore, the fine tuning of *SMG11* expression will be a promising strategy for increasing grain size and improving grain yield in rice.

## Conclusions

The rice *smg11* mutant shows small grains, dense panicles and the increased grain number. The *smg11* is a new allele of *DWARF2* (*D2*), which encodes a cytochrome P450 (CYP90D2). The *SMG11* controls grain size by promoting cell expansion in grain hulls. *SMG11* regulates cell expansion, at least in part, by influencing expression of several grain size genes involved in the regulation of cell expansion, revealing a novel link between *D2/SMG11* and known grain size genes. The suitable expression of *SMG11* increases grain size, grain weight and grain yield in rice. Our findings define the functions of *D2/SMG11* in grain size and grain yield, suggesting that an optimized expression of *D2/SMG11* is a promising approach to improve grain yield in rice.

## Methods

### Plant Materials and Growth Conditions

The *smg11* mutant was isolated from the *japonica* variety Kuanyejing (KYJ) mutagenized with EMS. Rice plants were grown in the experimental fields of Hainan (Lingshui, Hainan), the China National Rice Research Institute (Hangzhou, China) and Institute of Genetics and Developmental Biology (Beijing, China) under natural growth conditions.

### Morphological and cellular analysis

Mature grains were observed under a Leica microscope (LEICA S8APO; Leica microsystems, Wetzlar, Germany) and photographed using a Leica CCD (DFC420). Grain length and width were measured using Image J software. Grain weight was determined by weighting 1000 dry grains using an electronic analytical balance (Mettler Toledo AL104, China). The weights of three replicates were measured for each grain lot.

The size of epidermal cells in spikelet hulls were investigated using a scanning electron microscope. The samples were fixed in FAA solution (glacial acetic acid: formalin: 50% ethanol; 1:1:18) at 4 °C overnight, dehydrated in a graded ethanol series, and substituted with 100% ethanol. The critical-point drier (HITACHI HCP-2) was used to dry samples. The samples were dissected under a microscope (LEICA S8APO; Leica microsystems, Wetzlar, Germany), sputter-coated with platinum and observed using a scanning electron microscope (HITACHI S-3000 N; Hitachi High-Technologies Corporation, Tokyo, Japan). Cell size was measured using Image J software.

### Molecular cloning of *D2/SMG11*

To identify *smg11* mutation, we generated an F2 population from a cross between *smg11* and KYJ. We selected plants with *smg11* phenotypes from this F2 population and pooled their genomic DNA for the whole genome sequencing. We obtained a total of 5.6 Gbp of short reads for KYJ and 8.7 Gbp for the bulked F2. Then these short reads were alignment to the reference genome sequence (Nipponbare), and 2928 SNPs and 423 INDELs which are specific for the bulked F2 were identified. In these SNP/INDELs position, sequence of KYJ is same to Nipponbare. Thus, these SNP/INDELs represent the polymorphism between the bulked F2 and KYJ. For these SNPs and INDELs, the SNP/INDEL-index (the ratio between the number of reads of a mutant SNP/INDEL and the total number of reads corresponding to the SNP/INDEL) were calculated. The causative SNP/INDEL should be shared by all the mutant F2 plants and therefore has a SNP/INDEL-index = 1. Among them, 47 SNPs and 34 INDELs have a SNP/INDEL-index = 1, while only 2 SNPs were identified in extron. The SNP1 represents a missense mutation of a proline (CCC) codon to a leucine (CTC) in *LOC_Os01g10040*, while the SNP2 is corresponded to a synonymous mutation. Thus, these results suggested that *LOC_Os01g10040* might represent the *SMG11* gene.

### Plasmid construction and plant transformation

The *pActin: SMG11* construct was conducted using a PCR-based Gateway system. The *SMG11/D2* gene was amplified using the primers SMG11-F and SMG11-R (Additional file 2: Table S1). PCR products were subcloned into the *pCR8/GW/TOPO TA* cloning vector (Invitrogen, Carlsbad, CA, USA). The *SMG11* gene was further cloned into the *pIPKb003* vector with the *Actin* promoter to generate the plasmid *pActin: SMG11*. The *pActin: SMG11* plasmid was introduced into *Agrobacterium* strain EHA105, and Zhonghua 11 (ZH11) and *smg11* were transformed according to a previous method (Hiei et al., 1994).

### RNA extraction and quantitative real-time RT-PCR

Total RNA was isolated from young panicles of KYJ and *smg11* using plant RNeasy Mini Kit according to the manufacture manual (TIANGEN, Beijing, China). RNAs were quantified and reversely transcripted into cDNA using SuperScript III Reverse Transcriptase (Invitrogen). First-strand cDNA was synthesized from 3 μg of total RNAs. Reverse transcription was performed at 50 °C for 1 h and 70 °C for 15 min. Quantitative real-time RT-PCR analysis was performed using the CFX96 Real-Time PCR detection system (Bio-Rad) and SYBR Green I Master (Roche, Mannheim, Germany). *ACTIN1* was used as an internal control. The primers used in quantitative real-time RT-PCR were described in Additional file 2: Table S1.

## Additional Files

Additional file 1: Figure S1. Identification of the *smg11* mutation. **Figure S2.** Overexpression of *SMG11* complements the phenotypes of *smg11*. **Figure S3**. Alignment of D2/SMG11 homologs in rice and Arabidopsis. **Figure S4.** Effect of *SMG11* on cell number in spikelet hulls. **Figure S5**. *smg11* influences leaf angle. **Figure S6.** Exogenous application of BL rescues the leaf angle phenotype of *smg11*. (PDF 352 kb)
Additional file 2: Table S1. Primers used in this study (DOCX 19 kb)
