# Supplementary material for: SMALL GRAIN 11 Controls Grain Size, Grain Number and Grain Yield in Rice
Source: Rice (N Y). 2016 Nov 29;9:64. doi: 10.1186/s12284-016-0136-z (PMC5127926; doi:10.1186/s12284-016-0136-z)
Supplement: Additional file 1: Figure S1. — Identification of the smg11 mutation. Figure S2. Overexpression of SMG11 complements the phenotypes of smg11. Figure S3. Alignment of D2/SMG11 homologs in rice and Arabidopsis. Figure S4. Effect of SMG11 on cell number in spikelet hulls. Figure S5. smg11 influences leaf angle. Figure S6. Exogenous application of BL rescues the leaf angle phenotype of smg11. (PDF 352 kb) [file 12284_2016_136_MOESM1_ESM.pdf]

No.	Chromosome	Position	Reference	Alteration	Gene	Note
SNP1	Chr1	5236843	C	T	LOC_Os01g10040	nonsynonymous
SNP2	Chr1	5655212	C	T	LOC_Os01g10590	synonymous

Figure S1. Identification of the *smg11* mutation.
The whole genome sequencing reveals the SNP1 and SNP2 in the gene regions that are associated with the *smg11* phenotype.

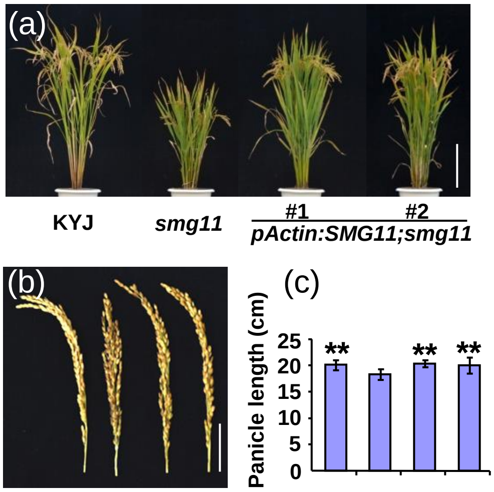

Figure S2. Overexpression of *SMG11* complements the phenotypes of *smg11*.

(a) Plants of KYJ, *smg11*, *pActin:SMG11;smg11#1* and *pActin:SMG11;smg11#2*. *pActin:SMG11;smg11* is *smg11* transformed with *pActin:SMG11*.

(b) Panicles of KYJ, *smg11*, *pActin:SMG11;smg11#1* and *pActin:SMG11;smg11#2*.

(c) Panicle length of KYJ, *smg11*, *pActin:SMG11;smg11#1* and *pActin:SMG11;smg11#2*. .

Values (c) are given as mean ± SD. **P < 0.01 compared with *smg11* by Student's t-test.

Bars: 20cm in (a), 5 cm in (b).

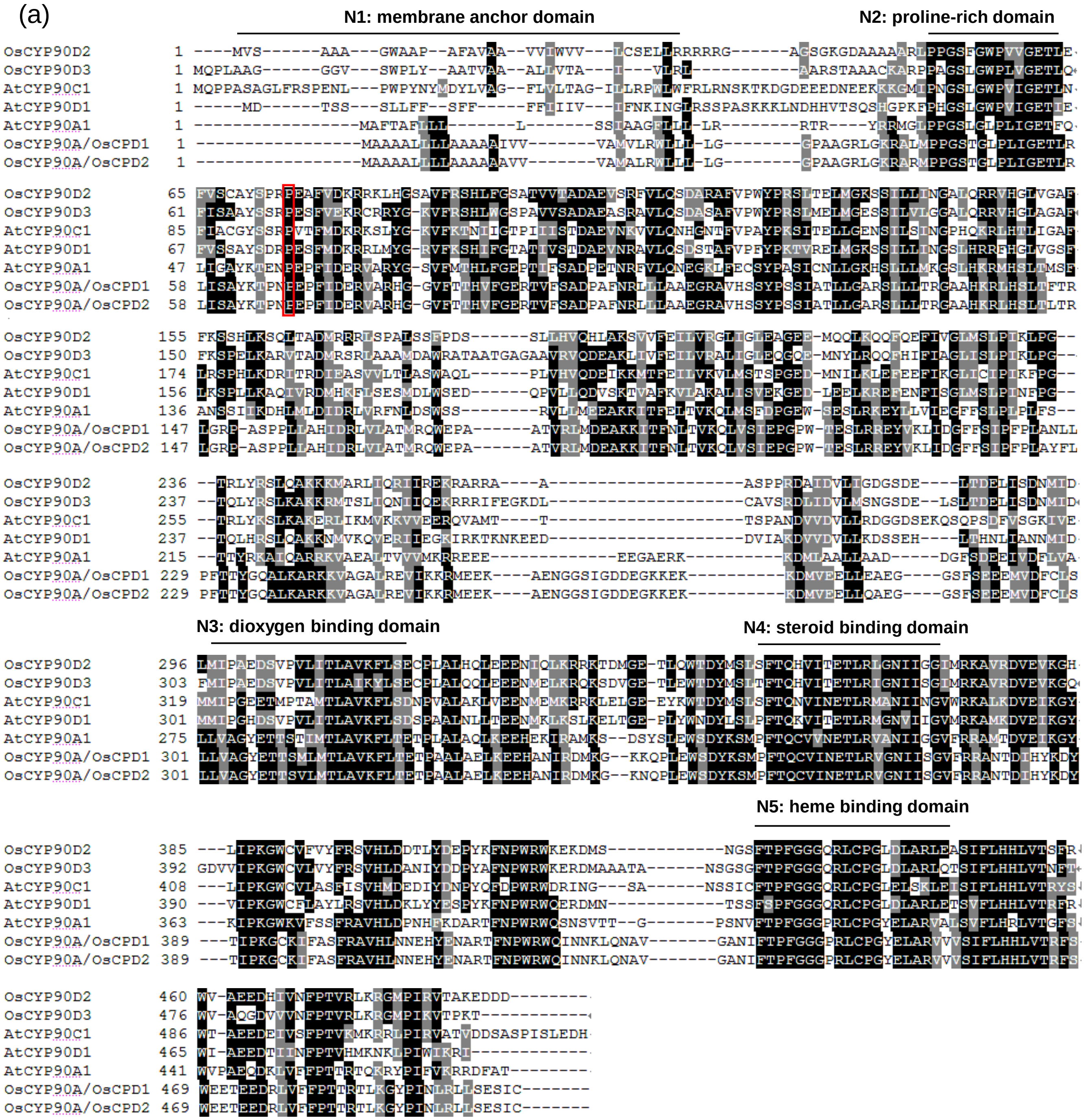

Figure S3. Alignment of D2/SMG11 homologs in rice and Arabidopsis.

The red box shows the conserved amino acid (Pro) at the position 74. The predicted domains are shown.

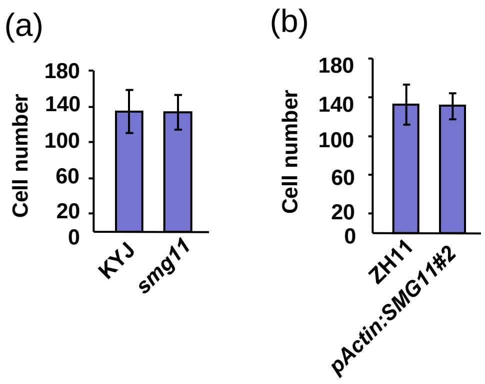

Figure S4. Effects of *SMG11* on cell number in spikelet hulls.
(a) The calculated number of outer epidermal cells in the grain-length direction in KYJ and *smg11* lemmas.
(b) The calculated number of outer epidermal cells in the grain-length direction in ZH11 and *pActin:SMG11#2* lemmas.

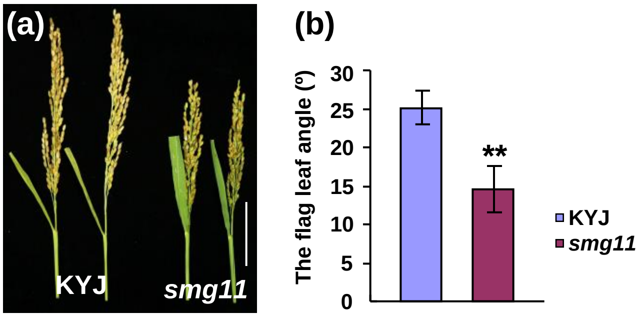

Figure S5. *smg11* influences leaf angle.

(a) Comparison of KYJ and *smg11* lamina joints at the mature stage.

(b) The flag leaf angle of KYJ and *smg11* plants at the mature stage.

Values (b) are given as mean ± SD. **P < 0.01 compared with KYJ by Student's t-test.

Bars: 5cm in (a).

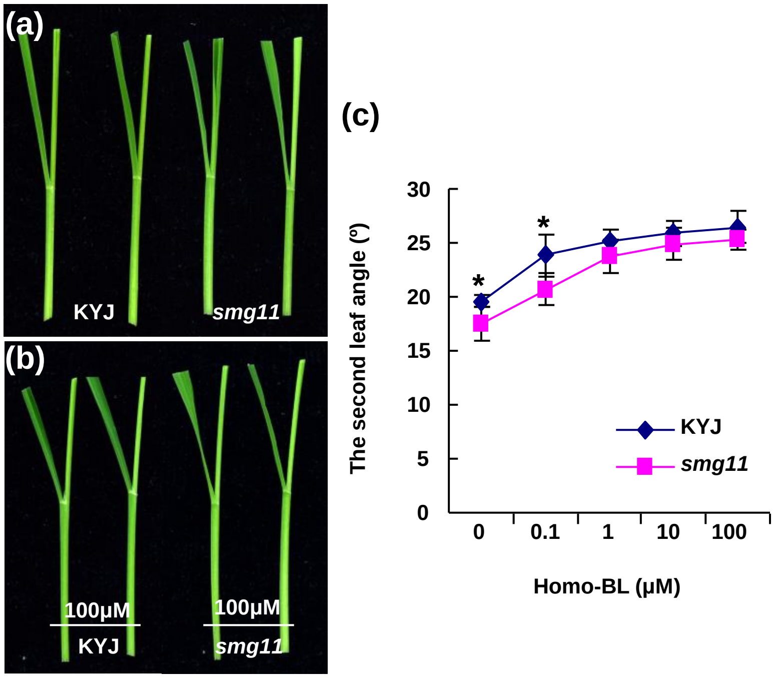

Figure S6. Exogenous application of BL rescues the leaf angle phenotype of *smg11*.
(a) The second leaf joint of KYJ and *smg11* seedlings without homo-BL (in the trefoil stage).
(b) The second leaf joint of KYJ and *smg11* seedlings with 100 μM homo-BL (in the trefoil stage).
(c) The second leaf angle of KYJ and *smg11* treated by different concentrations of homo-BL.
Values (c) are given as mean ± SD. *P < 0.05 compared with KYJ by Student's t-test.
