# Supplementary material for: SMALL GRAIN 11 Controls Grain Size, Grain Number and Grain Yield in Rice
Source: Rice (N Y). 2016 Nov 29;9:64. doi: 10.1186/s12284-016-0136-z (PMC5127926; doi:10.1186/s12284-016-0136-z)
Supplement: Additional file 2: Table S1. — Primers used in this study (DOCX 19 kb) [file 12284_2016_136_MOESM2_ESM.docx]

**Table S1 Primers used in this study.**

| **Primer Name** | **Primer sequence** |
| --- | --- |
| **Primers for RT-PCR and QRT-PCR** | |
| *ACTIN1*-F | TGCTATGTACGTCGCCATCCAG |
| *ACTIN1*-R | AATGAGTAACCACGCTCCGTCA |
| *DEP1*-F | CCGTTTCTCGTTCTGGAT |
| *DEP1*-R | ATCTGTGCCTCCTTCTCT |
| *DEP2*-F | TGCGTGATAGCCTAGAACGAAG |
| *DEP2*-R | CTGGAATCAGCACTCCTGGATG |
| *DEP3*-F | TGGTGGACCAAGCTGTATCA |
| *DEP3*-R | ATCACCTTCTTCCCCCTGAA |
| *APG*-F | GCGTCATGAACTTCACCTTCTTCTC |
| *APG*-R | ATCGACATCATCTGCACCTGCA |
| *PGL2*-F | ATGTCGAGCAGAAGGTCGTC |
| *PGL2*-R | TCAGGAGCGGAGGATGCTGC |
| *PGL1*-F | GGGGTACCATGTCAAGCCGGAGGTCAC |
| *PGL1*-R | CGGGATCCCTACATCAGAAGGCTGCGGA |
| *SRS3*-F | CTCTTCTATGGAACCTGACAG |
| *SRS3*-R | CTGAGAAGCTGAAGCAGATG |
| *SRS5*-F | ATGAGGGAGTGCATCTCGAT |
| *SRS5*-R | CAAGATCGACGAAGACAGCA |
| *GS2*-F | ATTCCAAGTACTGCGAGCGC |
| *GS2*-R | GGCGACCAGCTGCGTTT |
| *GL7-S1*-F | GAGGCCACAGGGAAGATGCAA |
| *GL7-S1*-R | GGAATATTTATATTTCTTCTATAGTTTAATCTTCA |
| *SPL13-F* | AACCCGCCGTTCCAGATCAG |
| *SPL13-R* | AAGAAGGGACGTAGGTGGTG |
| *DWARF11*-F | TGGCGATGACATTCCGATG |
| *DWARF11*-R | GCAACTGCAAACCTGTCAGGA |
| *OsBRD1*-F | TCAACCTTCCTGGAACCAAC |
| *OsBRD1*-R | TCTGTGAGCTTCTCCCTGGT |
| *DWARF4*-F | AGTCGCGTGCTGCCATTCT |
| *DWARF4*-R | AGCTCAGCA AGAGGTCCAGGAT |
| *D61*-F | GTTGGACGGCCTTACGTTTATC |
| *D61*-R | GCTGGTAAACTCCAGCAAGC |
| *OsBZR1*-F | GACAACAACGAGGTGCTCAA |
| *OsBZR1*-R | GCTTACATCCCTTGCGGTAG |
| *GSK2*-F | CTCCTTGGTCAGCCATTGTT |
| *GSK2*-R | GCGAGGTCTATTGCTTCAGG |
| *BU1*-F | GTAGCCAGCTTGATCTCATCTC |
| *BU1*-R | GGGACGACTCTACTGCATCA |
| *DLT* -F | TGCGGATACTCAACGCCATCA |
| *DLT* -R | ACTCGCCGACTCCGGTGATC |
| *GS3* -F | CATCGGAGAAGCGAAGTCAT |
| *GS3* -R | TTGAGGTTGAAGGAGGAGGA |
| *GW2* -F | CAGCAGCGCATTCCCAGTTTTC |
| *GW2* -R | GTGGTCAGCCGAGCACTCTC |
| *GS5* -F | AGTGGACTGCTTCCAGGGAAG |
| *GS5* -R | CACGCAGTACCGAGAACTGA |
| *GL3* -F | TCACAACTCCCAGGATAGG |
| *GL3* -R | TTTGTCTCGCTCGCTCAT |
| *GW8* -F | AGGAGTTTGATGAGGCCAAG |
| *GW8* -R | GCGTGTAGTATGGGCTCTCC |
| **Primers for the CAPS marker** | |
| dCAPS1-F | TGGAGTTCGTGTCGTGCGCCTAC |
| dCAPS1-R | CGTCCGCCGTCACCACCGT |
| **Primers for constructs** | |
| *SMG11* -F | ATGGTGTCGGCGGCCGCCGGTT |
| *SMG11* -R | CTAGTCGTCGTCCTCCTT |
